# *OPRM1* A118G polymorphism modulating motor pathway for pain adaptability in women with primary dysmenorrhea

**DOI:** 10.3389/fnins.2023.1179851

**Published:** 2023-06-12

**Authors:** Pei-Shan Hsu, Chou-Ming Cheng, Hsiang-Tai Chao, Ming-Wei Lin, Wei-Chi Li, Lin-Chien Lee, Ching-Hsiung Liu, Li-Fen Chen, Jen-Chuen Hsieh

**Affiliations:** ^1^Institute of Brain Science, College of Medicine, National Yang Ming Chiao Tung University, Taipei, Taiwan; ^2^Integrated Brain Research Unit, Division of Clinical Research, Department of Medical Research, Taipei Veterans General Hospital, Taipei, Taiwan; ^3^Department of Chinese Medicine, Taipei Tzu Chi Hospital, Buddhist Tzu Chi Medical Foundation, New Taipei City, Taiwan; ^4^Department of Obstetrics and Gynecology, Taipei Veterans General Hospital, Taipei, Taiwan; ^5^Institute of Public Health, National Yang Ming Chiao Tung University, Taipei, Taiwan; ^6^Department of Biological Science and Technology, College of Biological Science and Technology, National Yang Ming Chiao Tung University, Hsinchu, Taiwan; ^7^Department of Physical Medicine and Rehabilitation, Cheng Hsin General Hospital, Taipei, Taiwan; ^8^Department of Neurology, Lotung Poh-Ai Hospital, Yilan, Taiwan; ^9^Brain Research Center, National Yang Ming Chiao Tung University, Taipei, Taiwan; ^10^Institute of Biomedical Informatics, College of Medicine, National Yang Ming Chiao Tung University, Taipei, Taiwan; ^11^Center for Intelligent Drug Systems and Smart Bio-devices, National Yang Ming Chiao Tung University, Hsinchu, Taiwan

**Keywords:** primary dysmenorrhea, *OPRM1* A118G polymorphism, white matter, diffusion tensor imaging, descending pain modulatory systems, motor pathway

## Abstract

**Introduction:**

Primary dysmenorrhea (PDM) is a common condition among women of reproductive age, characterized by menstrual pain in the absence of any organic causes. Previous research has established a link between the A118G polymorphism in the mu-opioid receptor (*OPRM1*) gene and pain experience in PDM. Specifically, carriers of the G allele have been found to exhibit maladaptive functional connectivity between the descending pain modulatory system and the motor system in young women with PDM. This study aims to explore the potential relationship between the *OPRM1* A118G polymorphism and changes in white matter in young women with PDM.

**Methods:**

The study enrolled 43 individuals with PDM, including 13 AA homozygotes and 30 G allele carriers. Diffusion tensor imaging (DTI) scans were performed during both the menstrual and peri-ovulatory phases, and tract-based spatial statistics (TBSS) and probabilistic tractography were used to explore variations in white matter microstructure related to the *OPRM1* A118G polymorphism. The short-form McGill Pain Questionnaire (MPQ) was used to access participants’ pain experience during the MEN phase.

**Results:**

Two-way ANOVA on TBSS analysis revealed a significant main effect of genotype, with no phase effect or phase-gene interaction detected. Planned contrast analysis showed that during the menstrual phase, G allele carriers had higher fractional anisotropy (FA) and lower radial diffusivity in the corpus callosum and the left corona radiata compared to AA homozygotes. Tractographic analysis indicated the involvement of the left internal capsule, left corticospinal tract, and bilateral medial motor cortex. Additionally, the mean FA of the corpus callosum and the corona radiata was negatively correlated with MPQ scales in AA homozygotes, but this correlation was not observed in G allele carriers. No significant genotype difference was found during the pain-free peri-ovulary phase.

**Discussion:**

*OPRM1* A118G polymorphism may influence the connection between structural integrity and dysmenorrheic pain, where the G allele could impede the pain-regulating effects of the A allele. These novel findings shed light on the underlying mechanisms of both adaptive and maladaptive structural neuroplasticity in PDM, depending on the specific *OPRM1* polymorphism.

## 1. Introduction

Primary dysmenorrhea (PDM) is a common gynecological disorder among young women characterized by menstrual pain in the absence of any observable pelvic abnormalities (Habibi et al., 2015; Iacovides et al., 2015). PDM is primarily caused by uterine myometrial hypercontractility and vasoconstriction, which can be attributed to various factors such as an increase in prostaglandin, cytokines, and vasopressin (Berkley, 2013; Ferries-Rowe et al., 2020). In addition, PDM can be linked to abnormal pain control mechanisms, as evidenced by structural and functional changes in pain processing networks (Berkley, 2013; Low et al., 2018). PDM is often associated with functional pain disorders and chronic pain conditions such as irritable bowel syndrome, fibromyalgia, chronic fatigue syndrome, and lower back pain in adulthood (Altman et al., 2006; Berkley, 2013; Chung et al., 2014; Tu et al., 2020). It is suggested that maladaptive changes in the descending pain modulation system (DPMS) in young women with PDM may contribute to the high incidence of comorbidity with functional pain disorders in later life (Wei et al., 2016a).

During menstruation, individuals with PDM have been found to experience pelvic floor hypersensitivity (Iacovides et al., 2015; Lima et al., 2019). Pelvic muscle training, such as Kegel exercises, has demonstrated positive effects on managing pelvic pain of various types, including PDM (Ortiz et al., 2015; ElDeeb et al., 2019; Scott et al., 2020). It has been reported that the representation of the pelvic floor muscle in the motor cortex involves overlapping areas of the medial primary motor cortex (M1) and the supplementary motor area (SMA) (Yani et al., 2018). To define these regions of interest in our study, we referred to them as the medial motor cortex (MMC), which includes the medial M1 and pre-SMA/SMA regions. Recent research has suggested a possible link between dysmenorrhea and motor cortex dysfunction (Kutch and Tu, 2016). Our previous study has also revealed abnormal functional connectivity between the periaqueductal gray (PAG) and MMC in young women with PDM implicating possible dysregulation of the motor system and DPMS (Wei et al., 2016b). Therefore, it is crucial to investigate further the relationship between sensorimotor representation and pelvic pain processing in PDM.

The substitution of adenine with guanine at codon 118 (A118G) in the mu-opioid receptor (OPRM1) gene results a single nucleotide polymorphism (SNP) that has been associated with decreased OPRM1 expression (Zhang et al., 2005), heightened pain sensitivity (Yao et al., 2015), and increased analgesic use (Sia et al., 2008). OPRM1 is responsible for the pain-reducing effects of opioids within the central nervous system, and individuals carrying the G allele may be at a higher risk for developing chronic pain (Fields, 2004). According to our previous study using functional magnetic resonance imaging (fMRI), there is evidence to suggest that variations in pain perception and neural regulation in individuals may be attributed to differences in the *OPRM1* genotype, specifically affecting the functional connectivity between the sensorimotor and DPMS brain regions (Wei et al., 2017). The study found that active cortical modulation may be present during menstrual pain and that this may explain why AA homozygotes rated their pain experience lower than G allele carriers. Additionally, studies have suggested that white matter properties in the brain may predict pain chronification (Mansour et al., 2013). However, the current relationship between *OPRM1* A118G polymorphism and white matter changes in women with PDM is currently unknown.

Neuroimaging alterations during menstruation (painful state) were regarded as state changes, whereas alterations during the peri-ovulatory phase (pain-free state) were regarded as trait changes (Wei et al., 2016a). Specifically, our voxel-based morphometric study of gray matter volume found that PDM may be associated with cyclic state changes during the menstrual phase (MEN) in PDM subjects (Tu et al., 2013). Furthermore, our resting state fMRI-functional connectivity study revealed that only the G allele carriers of PDM subjects, compared to controls, may have hyperconnectivity in the PAG- MMC network during the MEN, implicating subclinical dysregulated pain modulation (Wei et al., 2017). This dysfunctional DPMS involving the MMC and PAG is a common factor in many chronic pelvic pain disorders (Kutch and Tu, 2016; Wei et al., 2017).

Our investigation aimed to assess the potential link between white matter alterations and the *OPRM1* A118G polymorphism in individuals with PDM, utilizing diffusion tensor imaging (DTI) (Le Bihan et al., 2001; Nucifora et al., 2007). We employed the tract-based spatial statistics (TBSS) method and probabilistic tractography (Behrens et al., 2003a) to analyze white matter connectivity. TBSS is a voxel-wise, data-driven approach that allows for the calculation of DTI metrics in white matter tracts. Our study specifically focused on investigating whether the *OPRM1* A118G polymorphism is associated with state or trait changes in white matter connectivity in individuals with PDM, with an emphasis on the connectivity between the motor cortex (particularly MMC) and DPMS.

## 2. Materials and methods

### 2.1. Subjects

The participants were selected based on the following criteria: (1) a menstrual cycle of approximately 27–32 days, (2) right-handedness as determined by the Edinburgh Handedness Inventory, and (3) a history of menstrual pain lasting more than 6 months, with an average pain score greater than 4 on a 0–10 verbal numerical scale (VNS) for the past 6 months under routine management for those with PDM. Subjects were excluded if they met any of the following conditions: (1) use of any medications, contraceptives, or hormone supplements in the 6 months prior to the study, (2) pituitary gland disease, (3) organic pelvic disease, (4) psychiatric or neurological disorders, (5) head injury with loss of consciousness, (6) pregnancy or plans to conceive, (7) history of childbirth, (8) metal implants, pacemakers, claustrophobia, or any contraindications to MRI. Participants were not allowed to take analgesics 24 h before the experiment. All subjects with PDM were diagnosed by a gynecologist and underwent a pelvic ultrasound to rule out organic pelvic diseases.

### 2.2. Experimental design

Blood samples were collected at the outset of the study for genetic analysis, but the participants’ genotypes were kept unknown until the scanning session. The short-form McGill Pain Questionnaire (MPQ) (Melzack, 1987) was used to access participants’ pain experience during the MEN phase. Two MRI scans, including T1 and DTI images, were performed at two time points during the menstrual cycle: during menstruation (days 1–3, MEN phase) and during the periovulatory phase (days 12–16, POV phase). For further information on the genetic analysis, please refer to our published article (Wei et al., 2016b).

### 2.3. Image acquisition

The imaging data for all participants was collected using a 3.0 T MRI scanner (Magnetom Trio Tim, Siemens, Erlangen, Germany), located at the National Yang-Ming University. Diffusion weight image (DWI) was acquired using 30 different directions and a *b*-value of 900 s/mm^2^, in addition to a single *b*-value of 0 s/mm^2^ image. The imaging parameters for DWI were set as TR/TE = 7,900 ms/79 ms, bandwidth = 1,346 Hz/Px, 70 slices with a thickness of 2 mm and no interslice gaps, a field of view of 256 × 256 mm^2^, a matrix size of 128 × 128, and a voxel size of 2 × 2 × 2 mm^3^, with 3 excitations and an acquisition time of 13 min and 4 s. High-resolution T1-weighted images (T1WI) were obtained with the imaging parameters set as TR/TE = 2,530 ms/3.03 ms, inversion time = 1,100 ms, bandwidth = 130 Hz/Px, 192 slices with a thickness of 1 mm, a field of view of 224 × 256 mm^2^, a matrix size of 224 × 256, and a voxel size of 1 × 1 × 1 mm^3^, a flip angle of 7 degrees and an acquisition time of 5 min and 23 s.

### 2.4. Image preprocessing

The DTI images were processed using FMRIB Software Library (FSL) v5.0^1^ from the Oxford Center for Functional Brain MRI (Jenkinson et al., 2012). To perform the TBSS analysis, several steps were followed. First, the DTI images were corrected for eddy current distortion and movement, then registered to each participant’s corresponding b0 image with affine registration using the FMRIB Diffusion Toolbox (Andersson et al., 2007). Participants with head motion greater than 3 mm were excluded. The DWI runs were then averaged to improve the signal-to-noise ratio of the image. A binary brain mask of each subject was created using the individual average, and non-brain tissue was removed using the brain extraction tool (BET) (Smith, 2002). The DTIFIT function in FDT was used to fit the DTIs using a linear least square algorithm, generating DTI maps that assessed white matter integrity by measuring DTI metrics, including fractional anisotropy (FA), mean diffusivity (MD), radial diffusivity (RD), and axial diffusivity (AD). FA measures the difference between the largest eigenvalue and the other two and reflects the white matter microstructure. MD provides an average of all three eigenvalues and is sensitive to changes in cellularity, edema, and necrosis. An increase in RD, which is the average of the second and third eigenvalues, suggests demyelination in the white matter. AD, which only considers the first eigenvalue, tends to change with white matter pathology (Tromp and Scalars, 2016).

The standard TBSS procedure was then employed to analyze the results which included several steps (Smith et al., 2006). First, all subjects were aligned into a common space using a representative subject as the registration target. Non-linear alignment was performed on all FA images, and linear registration was performed on the MNI152 atlas template. The combined transformation was used to align all subjects’ FA images into the MNI152 space, creating a study-specific mean FA atlas. A skeletonized mean FA image was created by thinning all aligned FA images with a threshold of >0.2. The FA map of each subject was projected onto the FA skeleton by searching perpendicular to the local skeleton structure. Then voxel-wise statistics analysis across subjects was performed on the skeleton-space FA data. The other DTI metrics, including MD, RD, and AD, were evaluated in a similar way to the FA analysis to gain a deeper understanding of the brain’s microstructural integrity of subjects with PDM.

### 2.5. Statistical analyses and tractographic visualization

#### 2.5.1. Demographic information and psychophysiological measurements

The data analysis was conducted using GraphPad Prism 9 (version 9.1.1). As some of the psychophysiological data did not adhere to a normal distribution, a non-parametric analysis was employed, and the findings were presented as median (range). Statistical significance was considered when the value of *p* was less than 0.05. The chi-square test was utilized to examine the Hardy–Weinberg equilibrium of the *OPRM1* genotype distribution. The Mann–Whitney U test was utilized to examine the impact of genotype on demographic factors such as the Edinburgh Handedness Inventory score and MPQ scores.

#### 2.5.2. Image analysis and tractographic visualization

In the current study, a two-way ANOVA was used to examine the main effects and interactions of genotype and phase in the TBSS analysis of white matter microstructure. The FA skeleton map was analyzed using the FEAT function in FSL (Woolrich et al., 2004). Planned contrast methods were utilized to investigate genotype differences in each phase, with a two-sample *t*-test employed to detect subtle but potentially important findings (Wu and Slakter, 1990; Lee et al., 2018; Li et al., 2021). Non-parametric tests based on FSL permutation were used to compare the FA, with multiple comparisons corrected using the threshold-free cluster enhancement (TFCE) method at a significance level of *p* < 0.05 and a minimum cluster size of 30 voxels (Winkler et al., 2014). Additionally, the MD, RD, and AD metrics were also evaluated using TBSS procedure. The white matter label atlas of Johns Hopkins University-International Consortium for Brain Mapping (JHU-ICBM-DTI-81)^2^ was used to identify significant differences in white matter tracts as regions of interest (ROI). To facilitate better visualization, the thresholded TBSS images were enhanced to have a thicker appearance.

To confirm the location of TBSS clusters within the motor system fiber tracts, the study employed probabilistic tractography using a composite mask composed solely of all significant FA seeds, as previously described (Szabó et al., 2012; Borich et al., 2013). FA was selected as the primary metric because it provides a comprehensive measure of diffusivity and directionality, and holistically captures microstructure changes (Alexander et al., 2007; Tromp and Scalars, 2016). Tractography was initiated using the composite mask as the starting point, without utilizing any restricted waypoint or termination masks. BEDPOSTX in FDT was utilized to estimate the diffusion parameter, with two probabilistic fiber directions burned 900 times for tractography (Behrens et al., 2003b). The DTIs were registered to T1 and transformed into standard space (MNI 152) through nonlinear registration with FDT registration (Andersson et al., 2007). The final tractographic analysis included tracing 5,000 probabilistic streamlines from each voxel within the TBSS seed, using a curvature threshold of 0.2 and a step length of 0.5. The study combined data from all subjects using FSLeyes^3^ and applied a threshold of 5,000 for each subject, followed by a threshold of 300,000 streamlines for visualization.

#### 2.5.3. Correlation analysis between DTI metrics and pain behavior

To explore the connection between white matter plasticity and pain experience, we performed a Spearman’s correlation analysis on the FAs of the identified clusters. For each subject, we extracted the mean FA value from each ROI mask. The correlation between these values and MPQ scores was examined because the OPRM1 A118G polymorphism may affect the pain perception of individuals (Wei et al., 2017).

## 3. Results

### 3.1. Subjects

Participants were sourced through internet advertisements. Hundred and ten subjects with primary dysmenorrhea meet the inclusion criteria and were enrolled initially. Six participants were excluded from the study due to the presence of secondary dysmenorrhea, as detected by a pelvic ultrasound exam conducted by the gynecologist (HTC). Twelve subjects were excluded due to incidental abnormal brain findings identified in their MRI scans, and 35 subjects declined to participate. The final sample size consisted of 57 PDM patients who completed the two-phase study that involved behavioral assessments and neuroimaging scans.

**Figure 1:**
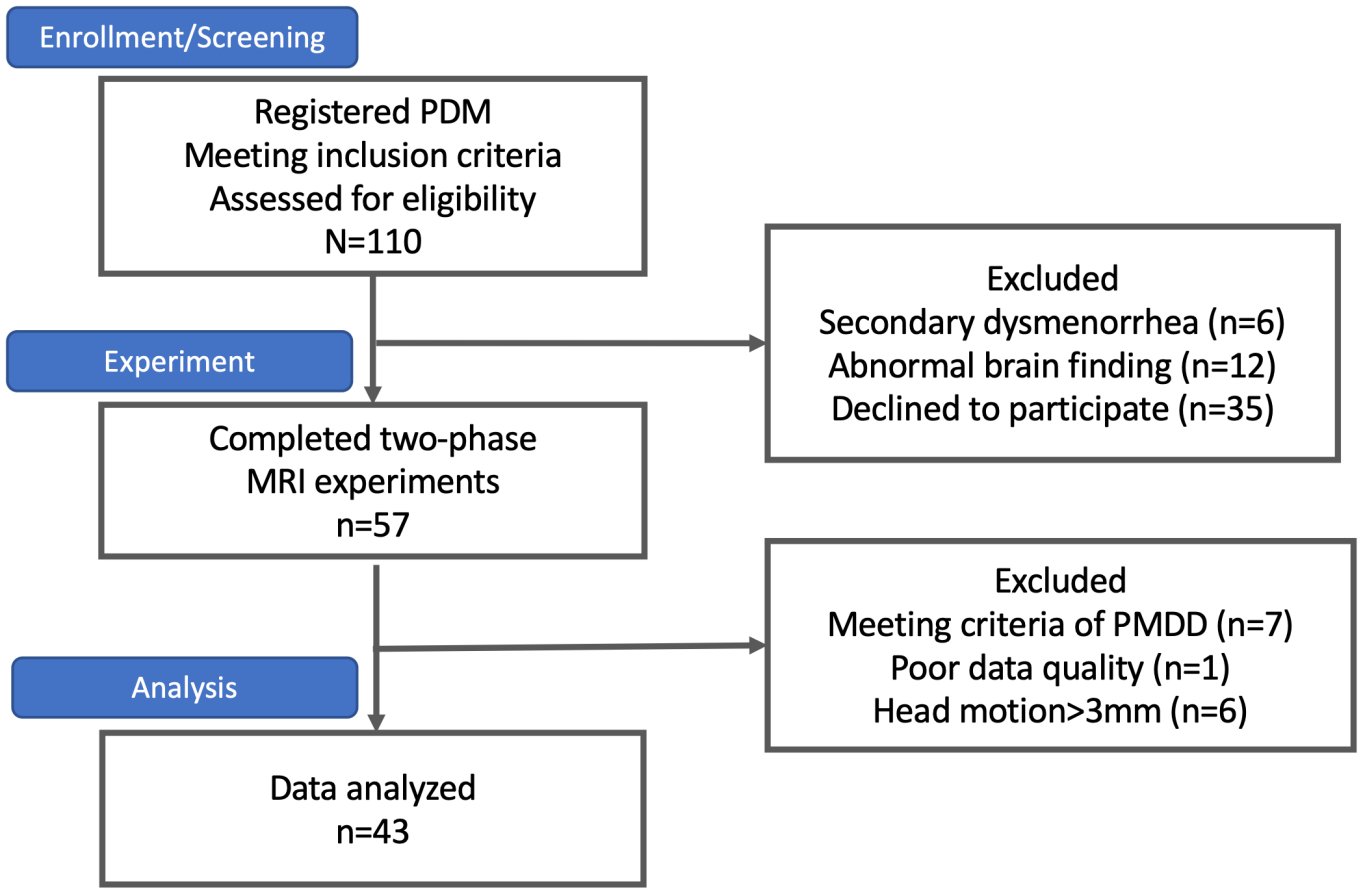
The subject flowchart. At the outset, 110 subjects with primary dysmenorrhea were enrolled, as indicated in the flowchart. However, six subjects were excluded due to secondary dysmenorrhea, 12 were excluded due to abnormal brain findings, and 35 declined to participate. Only 57 subjects successfully completed the two-phase study. Among them, seven subjects were excluded due to premenstrual dysphoric disorder (PMDD), one due to poor data quality, and six due to excessive head motion (>3 mm) during the scan. Consequently, the final sample size was 43 subjects.

Of these, 14 subjects with PDM were excluded further due to a high probability of premenstrual dysphoric disorder and disruption of daily life, poor data quality, or head motion greater than 3 mm during the scan. Finally, the study included 43 PDM patients (13 with AA genotype, 25 with AG genotype, and 5 with GG genotype, with a mean age of 23 [10] years) (Figure 1).

### 3.2. Genetic data and clinical characteristics

The distribution of the A118G gene in the PDM subjects (*p* = 0.38) did not deviate from the Hardy–Weinberg equilibrium. The AG heterozygotes and GG homozygotes were combined as G allele carriers, based on their similar clinical characteristics (Sia et al., 2008). There were no significant differences in demographic variables such as age, gynecological age, menstrual cycle, education, body mass index (BMI), or Edinburgh Handedness Inventory scores among the different genotypes (Table 1).

**Table 1:** Demographic data and baseline information.

	AA homozygotes (*n* = 13)	G allele carriers (*n* = 30)	Value of *p*
Calendar age (year)	23.0 (9.0)	23.0 (8.0)	0.27
Gynecological age (year)	12.0 (10.0)	10.0 (12.0)	0.07
Menstrual cycle (day)	29.5 (5.5)	29.0 (5.5)	0.69
Education (year)	16.0 (3.0)	16.0 (4.0)	0.27
BMI	20.6 (12.0)	20.5 (11.8)	0.20
Edinburg handedness	90.0 (60.0)	90.0 (60.0)	0.82
McGill pain (PRI_total)	32.0 (37.0)	34.0 (54.0)	0.87
PRI_sensory	16.0 (25.0)	18.0 (28.0)	0.55
PRI_affective	4.0 (10.0)	4.5 (11.0)	0.89
PRI_evaluation	5.0 (4.0)	5.0 (4.0)	0.56
PRI_miscellaneous	9.0 (12.0)	9.0 (15.0)	0.61
Present pain	3.0 (3.0)	3.0 (4.0)	0.82

Data are presented as median (range). The non-parametric Mann–Whitney *U* (two-tail) test was conducted for between-group comparisons. BMI, body mass index; PRI, pain rating index.

The subjects in the study had a long history of menstrual pain, with a median duration of 9 years (range 14 years), and pain lasting approximately 2 days per menstrual cycle (median [range] = 2 [4.9]). Over half of the subjects with PDM (55.8%) reported missing school or work due to their menstrual pain, and 44.18% of them required analgesics. However, there were no significant differences in the history of menstrual pain, duration of pain per cycle, or scores on the McGill Pain Questionnaire among the different genotypes (Table 1).

### 3.3. Differences in tract diffusion measurements

**Figure 2:**
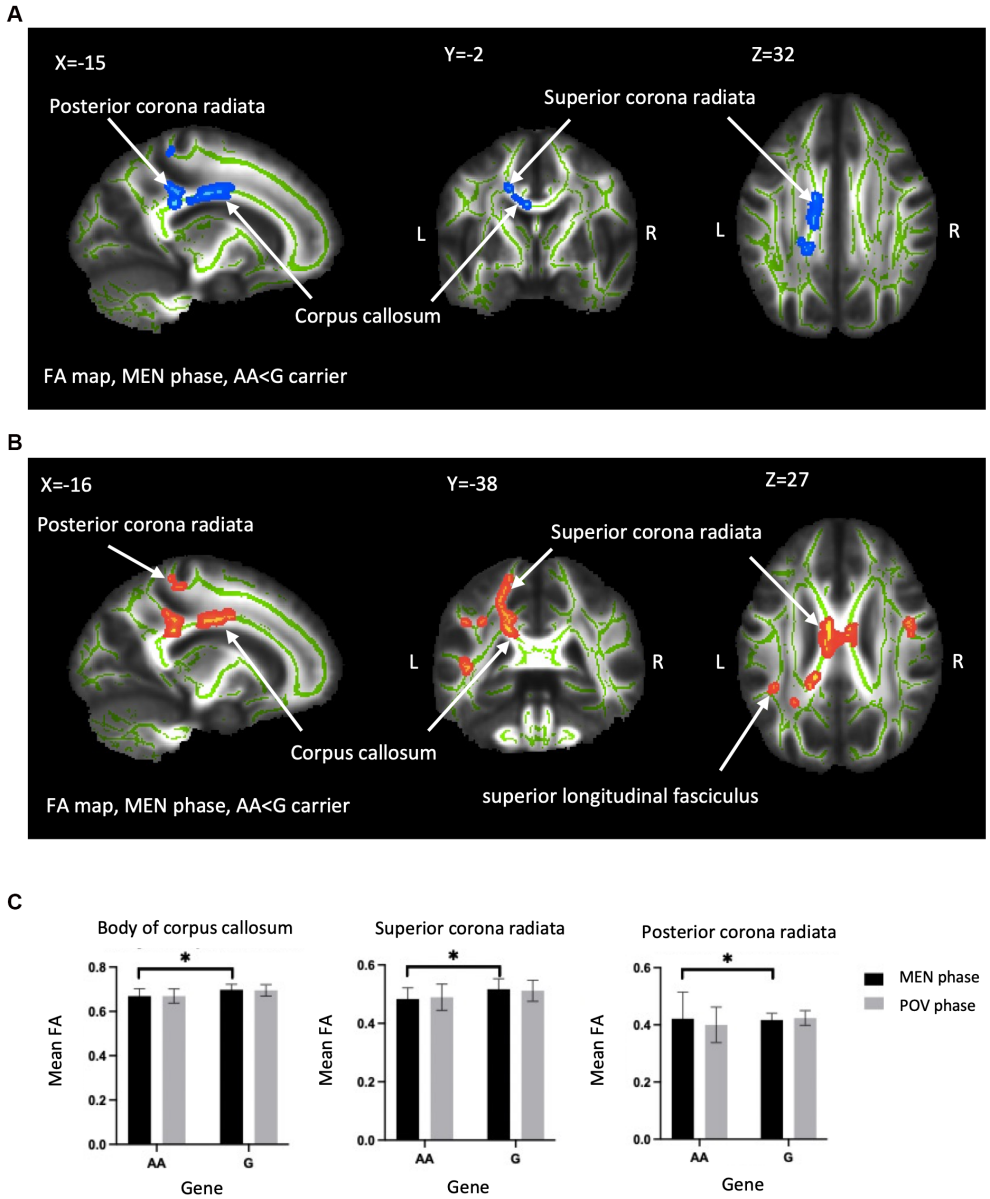
Between-genotype differences of DTI metrics. TBSS analyses showed group difference in FA and RD in the *OPRM1* A118G polymorphism during the MEN phase. **(A)** Blue regions indicate areas showing significantly lower FA in AA homozygotes compared to G carriers. **(B)** Red regions indicate significantly higher RD in AA homozygotes. The labeled clusters (white arrows) are significant at TFCE/FWE-corrected *p* < 0.05, cluster voxel >30, thickened for better visualization, and overlaid on the white matter skeleton (shown in green). The group differences are observed in the body of the corpus callosum, superior corona radiata, and posterior corona radiata. **(C)** The FA value (mean ± SD) of significant regions extracted from **(A,B)** for visualization. FA, fractional anisotropy; RD, radial diffusivity; MEN, menstrual; TFCE, threshold-free cluster enhancement; FWE, family-wise error; SD, standard deviation; TBSS, tract-based spatial statistics; L, left; R, right; Asterisks (*) indicate significant difference by TBSS procedure.

**Figure 3:**
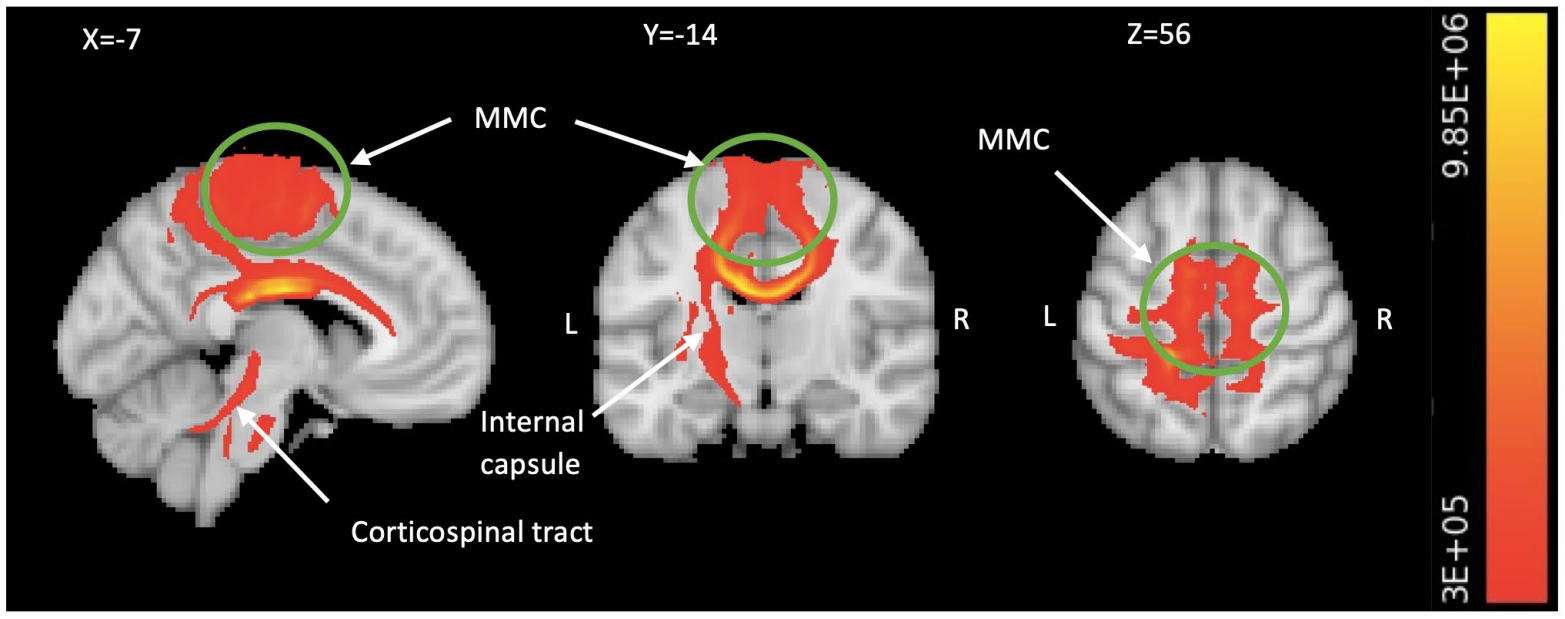
Motor system engagement revealed by TBSS-based tractography. The TBSS analysis generated probabilistic tractography (comprising all significant FA seeds in Table 2), which illustrates the connectivity between the bilateral MMC including medial M1 and pre-SMA/SMA (in green circle), all the way to the spinal cord *via* the corpus callosum, left internal capsule, and left corticospinal fiber pathways (in red/yellow). We visualize the result by aggregating data from all subjects and setting a threshold of 300,000 streamlines. The color bar indicates the number of streamlines traversing a voxel. TBSS, tract-based spatial statistics; FA, fractional anisotropy; MMC, medial motor cortex; M1, primary motor area; SMA, supplementary motor area; L, left; R, right.

A two-way ANOVA on TBSS analysis revealed a significant effect of genotype without any phase-gene interaction in PDM. Planned contrast analysis did not find any significant genotype differences (trait changes) during the POV phase. Compared to AA homozygotes, PDM individuals with the G allele displayed a state change during the MEN phase (planned contrast), characterized by increased FA and decreased RD in the corpus callosum (primarily in the body region and adjacent splenium) as well as the corona radiata (specifically the left superior and left posterior regions). All these regions are known to have projection fibers to the motor cortex (Hofer and Frahm, 2006; Park et al., 2008; Jang, 2009; Moeller et al., 2015). In addition, G allele carriers demonstrated decreased regional white matter RD in the left superior longitudinal fasciculus, which is thought to contain projection fibers to the motor cortex (Janelle et al., 2022), compared to AA homozygotes (Table 2 and Figure 2). Probabilistic tractography analysis (FA seeds only) revealed the involvement of the left internal capsule, left corticospinal tract, and bilateral MMC (*cf.* Hofer and Frahm, 2006; Figure 3).

**Table 2:** Between genotype differences in TBSS analysis.

Cluster location		*x*	*y*	*z*	Value of *p*	Cluster size (voxel)
MEN phase, G allele carriers >AA homozygotes						
Body of corpus callosum	FA	–17	–9	36	0.044	312
Splenium of corpus callosum	FA	−13	−36	26	0.046	35
Left superior corona radiata	FA	−17	−9	37	0.044	85
Left posterior corona radiata	FA	−22	−32	30	0.046	79
MEN phase, AA homozygotes > G allele carriers						
Body of corpus callosum	RD	7	19	18	0.048	175
Splenium of corpus callosum	RD	−13	−36	26	0.048	45
Left superior corona radiata	RD	−18	−20	36	0.048	65
Left posterior corona radiata	RD	−19	−40	36	0.046	101
Left superior longitudinal fasciculus	RD	−43	−42	3	0.048	51
POV phase, G allele carriers >AA homozygotes						
NS						
POV phase, AA homozygotes > G allele carriers						
NS						

TBSS, tract-based spatial statistics; OPRM1, mu opioid receptor; POV, periovulatory phase; MEN, menstrual phase; FA, fractional anisotropy; RD, radial diffusivity. All clusters are significant at FWE-corrected *p* < 0.05, cluster voxel > 30.

### 3.4. Correlation analysis

Table 3 illustrates that among AA homozygotes, the study discovered a negative correlation between the mean FA of the body of corpus callosum and the pain rating index (affective), as well as a negative correlation between the mean FA of the left posterior corona radiata and the pain rating index (sensory) of McGill Pain Questionnaire. Moreover, the study observed a positive correlation between the mean RD of the body of corpus callosum and the pain rating index (affective) in AA homozygotes. However, no significant correlation was found between DTI metrics and MPQ scales in G allele carriers.

**Table 3:** Correlation between the TBSS results and the MPQ scores.

	White matter tract	Behavior data	Value of *p*	*r*
MEN phase, white matter regions of G allele carriers> AA homozygotes (mean FA)				
AA homozygotes	Body of corpus callosum	PRI_affective	0.0103	−0.7503
	Left posterior corona radiata	PRI_sensory	0.04	−0.6328
G-allele carriers	NS	NS	NS	NS
MEN phase, white matter regions of AA homozygotes >G allele carrier (mean RD)				
AA homozygotes	Body of corpus callosum	PRI_affective	0.0221	0.6930
G-allele carriers	NS	NS	NS	NS

TBSS, tract-based spatial statistics; MPQ, McGill Pain Questionnaire; MEN, menstrual phase; FA, fractional anisotropy; RD, radial diffusivity; PRI, pain rating index of MPQ; NS, non-significant.

## 4. Discussion

Our study depicts that the *OPRM1* A118G polymorphism subtly influences the white matter microstructure during the painful MEN phase, but not during the pain-free POV phase. Specifically, AA homozygotes and G carriers exhibit different state changes. The current findings are consistent with our previous study which found that individuals with PDM who carry the G allele have a maladaptive motor cortex and descending pain modulatory systems (Wei et al., 2017). The present study further demonstrated that G allele carriers with PDM have higher FA and lower RD in the corpus callosum and the left corona radiata during the menstrual phase, as compared to AA homozygotes. TBSS-tractography analysis showed that these differences involved the left internal capsule, left corticospinal tract, and bilateral MMC. However, in AA homozygotes, the mean FA of the corpus callosum and the corona radiata was negatively correlated with pain-related scales, which was not present in the G allele carriers. These results suggest that the *OPRM1* A118G polymorphism may play a critical role in modulating dysmenorrheic pain and that the neuromodulatory capacity of the A allele may be reduced in the G allele group. Such menstrual phase related rapid structural alterations is corroborated by our previous voxel-based morphometric study of gray matter volume in PDM (Tu et al., 2013).

The corpus callosum is involved in selective attention (Banich, 2003) and pain perception (Stein et al., 1989). Damage to the corpus callosum can result in somatosensory processing disorders, disrupted emotional regulation, and decreased working memory capacity (Luerding et al., 2008; Short et al., 2013; Kim et al., 2014; Fang et al., 2017). Numerous chronic pain conditions, such as pelvic pain (Woodworth et al., 2015), irritable bowel syndrome (Ellingson et al., 2013; Fang et al., 2017), low back pain (Kregel et al., 2015), temporomandibular disorder (Moayedi et al., 2012), migraine (Yuan et al., 2012; Coppola et al., 2020), and fibromyalgia (Kim et al., 2014), are associated with corpus callosum white matter abnormalities. Agenesis of the corpus callosum has been associated with changes in sensory processing, such as a higher pain tolerance and threshold for pain perception (Demopoulos et al., 2015). Moreover, the corpus callosum’s enhanced interhemispheric connectivity can modulate attentional capacity, enabling individuals to concentrate on a specific task while disregarding others (e.g., hypnotic analgesia) (Horton et al., 2004). The corpus callosum consists of transcallosal fibers that connect the bilateral sensorimotor and superior frontal cortices, with a larger proportion of these fibers targeting the premotor, supplementary motor, and primary motor areas (Paul et al., 2007). The motor cortex has been pinpointed as playing a crucial role in modulating pain processes in PDM according to our previous functional MRI study (Wei et al., 2016a). In our current tractographic analysis of the PDM group, we found that the left corona radiata extends to the MMC, which corresponds to the motor representation of the pelvic floor muscle (Yani et al., 2018). Dysfunction in these regions has been linked to chronic pelvic pain (Kutch and Tu, 2016), and persistent pelvic pain has been suggested to cause axonal reorganization of the corticospinal tract in this region (Huang et al., 2016). These findings collectively suggest that G allele carriers exhibit maladaptive changes in the corticospinal tract of the corona radiata.

The corona radiata is a projection of fibers to the internal capsule and corticospinal tract, which contains motor neurons responsible for voluntary fine muscle movements and posture. The observed negative correlation between pain experience and FA of the corona radiata in the AA homozygous group may be attributed to learned motor responses that aim to adapt or alleviate pelvic pain physically (Huang et al., 2016). White matter integrity changes in the corona radiata, internal capsule, or corticospinal tract have been observed in many chronic pain conditions (Moayedi et al., 2012; Ellingson et al., 2013; Moana-Filho et al., 2013). This corticospinal tract is responsible for descending pain modulation and mediates inhibitory and facilitatory influences on spinal nociceptive transmission (Fishman et al., 2009). Several animal studies indicate that therapeutic motor stimulation, including stimulation of the sensorimotor cortex, can modulate the nociceptive response by activating the C fiber of the dorsal horn (Rojas-Piloni et al., 2010). Clinical studies that use repetitive motor cortex stimulation techniques, including transcranial magnetic stimulation (TMS), motor cortex stimulation (MCS), and transcranial direct current stimulation (tDCS), for treating neuropathic pain also suggest that top-down modulation of the thalamus, basal ganglia, and PAG in the brainstem leads to descending inhibition of the spinal cord (Garcia-Larrea and Peyron, 2007). Studies have shown that elevated FA in the corona radiata is not only present in individuals with PDM but also in those with other chronic pelvic pain conditions (Kilpatrick et al., 2014; Farmer et al., 2015; Kutch et al., 2015; Huang et al., 2016). It has been suggested that corticospinal excitability is reduced in response to acute muscle pain as a protective mechanism against further injury (Burns et al., 2016).

Our research revealed that the *OPRM1* A118G polymorphism is associated with genetic differences in FA and RD in certain white matter tracts, suggesting that it may affect axonal structure and myelination. Alterations in white matter integrity can occur through changes in axonal structure, myelination, fiber organization, and branching (Basser and Jones, 2002; Song et al., 2002; Bammer, 2003; Apkarian et al., 2005). For instance, FA may reflect myelination in specific regions of interest, while RD may indicate changes in membrane permeability and myelination (Tromp and Scalars, 2016). Decreased FA and increased RD have been linked to several chronic pain conditions, indicating changes in axonal structure, branching, or fiber crossing (Ellingson et al., 2013; Farmer et al., 2015; Kregel et al., 2015). On the contrary, one study has reported that women with PDM have higher FA in the corpus callosum and corticospinal tract, which correlates with the duration of pain (Liu et al., 2016). Our findings indicate that G allele carriers have higher FA and lower RD compared to AA carriers in the corpus callosum and corona radiata in MEN phase. However, there were no significant differences in MD and AD between the gene groups, suggesting that neuro edema, necrosis, and prominent white matter pathology (Tromp and Scalars, 2016) are not involved in the structural modulation of the brain in PDM by the *OPRM1* A118G polymorphism. While G carriers have higher FA in the corona radiata, the strong correlation observed between MPQ scores and the FA of the corona radiata (as well as the RDs of other white matter tracts) in the AA homozygous group is diminished in the group of individuals carrying the G allele (Table 3), indicative of dysregulated pain modulation of white matter tract during the stressful pain in G allele carrier PDM subjects. The discrepancy in the aforementioned studies of chronic pain disorders and PDM (acute cyclic pain in nature) pinpoints that the genotype-informed brain imaging approach is important in elucidating mechanisms and clarifying the discrepancies in the neuroimaging study of different types of clinical pain.

Although opioid receptors are absent in the primary motor area, other brain regions such as the primary somatosensory area, basal ganglia, and brain stem (particularly the PAG) contain abundant opioidergic neurons (Peckys and Landwehrmeyer, 1999; Kibaly et al., 2019; Sjöstedt et al., 2020). The primary somatosensory area is responsible for pain intensity recognition and perception (Vierck et al., 2013), while the basal ganglia, traditionally known as a motor hub, also have an important role in pain processing due to the significant overlap between the basal ganglion network and the sensorimotor network (Figley et al., 2017). In addition, the PAG, a key component of the descending pain modulatory system, also contains opioidergic neurons (Linnman et al., 2012). It is plausible that the *OPRM1* polymorphism may directly or indirectly influence these hub regions involved in motor cortex-actuated descending pain modulation, leading to variations in pain experiences among individuals.

The exact cause of the observed left lateralized expression in the corona radiata and corticospinal tract in our study remains unknown. However, one possible contributing factor could be asymmetric opioid availability. Previous studies have suggested that mu-opioid receptors are more abundant in the right hemisphere (Kantonen et al., 2020), while the OPRM1 gene has greater expression in the left ventral horn of the spinal cord (Kononenko et al., 2017). Nonetheless, the lateralization of clinical pain processing is a multifactorial phenomenon that involves various factors (Roza and Martinez-Padilla, 2021). Therefore, the asymmetrical gene distributions and expressions identified in previous studies may only partly explain the lateralized findings in our research. To gain a more comprehensive understanding of this topic, further investigation is necessary.

The study has some limitations that need to be considered. Firstly, the sample size, particularly in the AA group, was relatively small, but the distribution was in accordance with the Hardy–Weinberg equilibrium. Secondly, the use of 30 diffusion-direction tensor images without correction for top-down eddy current artifacts could affect the precision of the results. Further research with larger sample sizes and incorporating up-to-date techniques to correct for these pitfalls would be valuable to validate these findings.

To sum up, our study sheds light on the significance of the *OPRM1* A118G polymorphism for modulating structural integrity and dysmenorrheic pain in subjects with PDM. Individuals with AA homozygosity demonstrate better pain adaptability within the motor cortex-related pain modulation system, whereas those carrying the G allele display maladaptive changes. These genetic differences in white matter structure may contribute to variations in pain susceptibility and potentially lead to the chronic pain later in life. Our results provide insight into the neuroplasticity of the central nervous system in PDM and underscore the need for personalized pain management approaches. These findings highlight the impact of the *OPRM1* A118G polymorphism on the microstructure of white matter in individuals with PDM and suggest potential avenues for future research.

## Data availability statement

The original contributions presented in the study are included in the article/supplementary material, further inquiries can be directed to the corresponding author.

## Ethics statement

The studies involving human participants were reviewed and approved by Institutional Review Board of Taipei Veterans General Hospital. The patients/participants provided their written informed consent to participate in this study.

## Author contributions

P-SH: conceptualization, investigation, formal analysis, validation, methodology, data curation, writing – original draft, and visualization. C-MC: formal analysis and validation. H-TC: investigation and resources. M-WL: methodology and resources. W-CL: investigation and data curation. L-CL: investigation and validation. C-HL: investigation and validation. L-FC: conceptualization, methodology, resources, and funding acquisition. J-CH: conceptualization, methodology, resources, funding acquisition, project administration supervision, and writing – review and editing. All authors contributed to the article and approved the submitted version.

## Funding

This work was supported by the Taipei Veterans General Hospital (V100D-001, V100D-001-1, V100D-001-2, and V101C-152), National Science and Technology Council (NSC 100-2314-B010-006-MY3, NSC 100-2629-B-010-001, NSC 102-2629-B-010-001, MOST 103-2321-B-010-020, MOST 106-2629-B-010-001-MY3, MOST 108-2314-B-010-001, MOST 109-2314-B-101-001-MY3, and MOST 109-2314-B-350-001), MOST 111-2314-B-A49-067, TVGH-NTUH joint research program (VN103-4, VN104-03, VN105-03), and the Aim for the Top University Plan of the Ministry of Education of National Yang-Ming University. The funders had no role in study design, data collection and analysis, the decision to publish, or manuscript preparation.

## Conflict of interest

The authors declare that the research was conducted in the absence of any commercial or financial relationships that could be construed as a potential conflict of interest.

## Publisher’s note

All claims expressed in this article are solely those of the authors and do not necessarily represent those of their affiliated organizations, or those of the publisher, the editors and the reviewers. Any product that may be evaluated in this article, or claim that may be made by its manufacturer, is not guaranteed or endorsed by the publisher.

## Footnotes

^1^ https://fsl.fmrib.ox.ac.uk/fsl/fslwiki/FslInstallation

^2^ https://identifiers.org/neurovault.image:1401

^3^ https://git.fmrib.ox.ac.uk/fsl/fsleyes/fsleyes
